# Gamabufotalin, a major derivative of bufadienolide, inhibits VEGF-induced angiogenesis by suppressing VEGFR-2 signaling pathway

**DOI:** 10.18632/oncotarget.6514

**Published:** 2015-12-09

**Authors:** Ning Tang, Lei Shi, Zhenlong Yu, Peipei Dong, Chao Wang, Xiaokui Huo, Baojing Zhang, Shanshan Huang, Sa Deng, Kexin Liu, Tonghui Ma, Xiaobo Wang, Lijun Wu, Xiao-Chi Ma

**Affiliations:** ^1^ College of Pharmacy, Academy of Integrative Medicine, Key Laboratory of Pharmacokinetic and Drug Transport of Liaoning, Dalian Medical University, Dalian, China; ^2^ Institute of Cancer Stem Cell, Dalian Medical University, Dalian, China; ^3^ College of Basic Medical Science, Dalian Medical University, Dalian, China; ^4^ Department of Pharmacy and Traditional Chinese medicine, Chinese People's Liberation Army 210 Hospital, Dalian, China

**Keywords:** gamabufotalin, angiogenesis, vascular endothelial growth factor (VEGF), vascular endothelial growth factor receptor 2 (VEGFR-2), aortic ring

## Abstract

Gamabufotalin (CS-6), a main active compound isolated from Chinese medicine *Chansu*, has been shown to strongly inhibit cancer cell growth and inflammatory response. However, its effects on angiogenesis have not been known yet. Here, we sought to determine the biological effects of CS-6 on signaling mechanisms during angiogenesis. Our present results fully demonstrate that CS-6 could significantly inhibit VEGF triggered HUVECs proliferation, migration, invasion and tubulogenesis *in vitro* and blocked vascularization in Matrigel plugs impregnated in C57/BL6 mice as well as reduced vessel density in human lung tumor xenograft implanted in nude mice. Computer simulations revealed that CS-6 interacted with the ATP-binding sites of VEGFR-2 using molecular docking. Furthermore, western blot analysis indicated that CS-6 inhibited VEGF-induced phosphorylation of VEGFR-2 kinase and suppressed the activity of VEGFR-2-mediated signaling cascades. Therefore, our studies demonstrated that CS-6 inhibited angiogenesis by inhibiting the activation of VEGFR-2 signaling pathways and CS-6 could be a potential candidate in angiogenesis-related disease therapy.

## INTRODUCTION

Angiogenesis is a complex process which includes endothelial cells (ECs) proliferation, migration, basement membrane degeneration and new tube formation. It is required for a variety of physiologic processes like development and reproduction. However, angiogenesis also plays a vital role in the growth and spread of cancer as an adequate blood supply, which is necessary for tumor growth and invasion in normal tissues [1–4]. It is known that endothelial cells are normally in a highly quiescent state, and crucial to maintain vascular homeostasis. But they can transform into an active proliferative state if stimulated by angiogenic factors from the tumors, then grow, migrate and form new blood vessels, which offer oxygen and nutrients to the tumors. Inducing angiogenesis is one of the hallmarks of cancer. New blood vessels infiltrate tumors, supplying them with oxygen and nutrients, and offer a route for tumor metastasis. Studies have shown that inhibition of ECs growth can block tumor angiogenesis effectively [5–7]. Moreover, agents that prevent the growth of a tumor's blood vessels facilitate the regression or dormancy of established tumors and anti-angiogenesis treatment has been an extremely promising form of cancer therapy [8]. Therefore, inhibiting angiogenesis may be an effective method for inhibiting tumor growth and metastasis.

Angiogenesis is tightly controlled by the balance of pro- and anti-angiogenic factors, such as vascular endothelial growth factor (VEGF), platelet-derived growth factor (PDGF), epidermal growth factor (EGF) and angiogenin, etc [9]. Imbalanced pro-angiogenesis stimulation would trigger pathologically angiogenic diseases such as ischemic heart failure as well as cancer progression. Among the pro-angiogenic factors, VEGF is well known to play an important part in tumor biology and more specifically, in the process of tumor angiogenesis, as its expression has been detected in various malignant human tumors, like breast, ovary, kidney, urinary bladder, brain, lung and gastrointestinal tract tumors [10]. VEGF is a family composed of five isoforms denominated VEGF-A, VEGF-B, VEGF-C, VEGF-D and placental growth factor (PlGF). Each of these factors exerts its activity through binding to three high-affinity transmembrane receptors (VEGFR1, VEGFR2 and VEGFR3), and then promote angiogenesis through its ability to stimulate the growth, migration and invasion of endothelial cells [11–15]. Current evidence suggests that the interaction between VEGF and VEGFR-1(Flt1) plays a minor role in angiogenesis, while VEGFR-2 (Flk1/KDR: VEGFR-2 also known as KDR in human and Flk1 in mouse) mediates the major angiogenic function of VEGF [16, 17]. VEGFR-2 is strongly auto-phosphorylated in tumors by elevated VEGF expression [18]. VEGF binds to specific transmembrane receptors on ECs and lymphatic vessels in endothelial cell [19, 20]. VEGFR-2 signaling activation induces the phosphorylation of various downstream signal transduction mediators, including phosphoinositide 3-kinase (PI3K)/AKT/mTOR, and mitogen-activated protein kinase (MAPK), which ultimately control processes critical to angiogenesis such as endothelial cell proliferation, migration and tubulogenesis [21–24]. Therefore, VEGF and VEGFR-2 become potential molecular targets for anti-angiogenic tumor therapy. Several approaches have been taken to block VEGF/VEGFR-2 signaling pathways, such as inhibition of endogenous VEGF release and prevention of VEGF from binding to VEGFR-2 [25].

In recent years, some studies have revealed that natural products which exist in the traditional Chinese medicines [26], have the potential properties in treating different types of cancers. Toad venom (called “*Chansu*” in China) is a product of the skin gland of toads such as *Bufo bufo gargarizans* etc [27, 28], which has been widely used as a cardiotonic, diuretic, anodyne and hemostatic agent [29]. *Chansu* has been used as significant anticancer agents, amending the life quality of cancer patients [30]. Recent experimental studies have demonstrated that *Chansu* and its active compounds exhibit significant anti-tumor activity via the inhibition of cell proliferation, induction of cell differentiation and apoptosis, disruption of the cell cycle, inhibition of angiogenesis, reversal of multidrug resistance, and regulation of the immune response [31]. A previous study suggests that *Chansu* could induce apoptosis of cancer cells, such as human gastric cancer and bladder cancer cells [32, 33]. Gamabufotalin (CS-6), a major bufadienolide isolated from *Chansu*, had the high content of 1.75%-5%, and significant anti-tumor activity. It has been found that it is used for anti-inflammatory, acesodyne and anti-neoplasti. Our previous studies also found that CS-6 suppressed human lung cancer A549 cell proliferation, migration and invasion [34]. However, potential influence of CS-6 in endothelial angiogenic activity is totally unknown.

In this study, we have investigated the effects of gamabufotalin (CS-6) on angiogenesis and characterized the underlying molecular mechanisms. The anti-angiogenesis properties of CS-6 were evaluated *in vitro* using human umbilical vein endothelial cells (HUVECs) proliferation, migration and tube formation assays, *ex vivo* by aortic ring assay and *in vivo* by matrigel plug assay mouse models. Our results exhibited that CS-6 inhibited VEGF-mediated angiogenesis in endothelial cells, which suggesting that CS-6 could be used as a potential anti-angiogenesis agent that targets VEGF/VEGFR-2 signaling pathways.

## RESULTS

### CS-6 inhibits VEGF-induced cell proliferation of HUVECs

**Figure 1:**
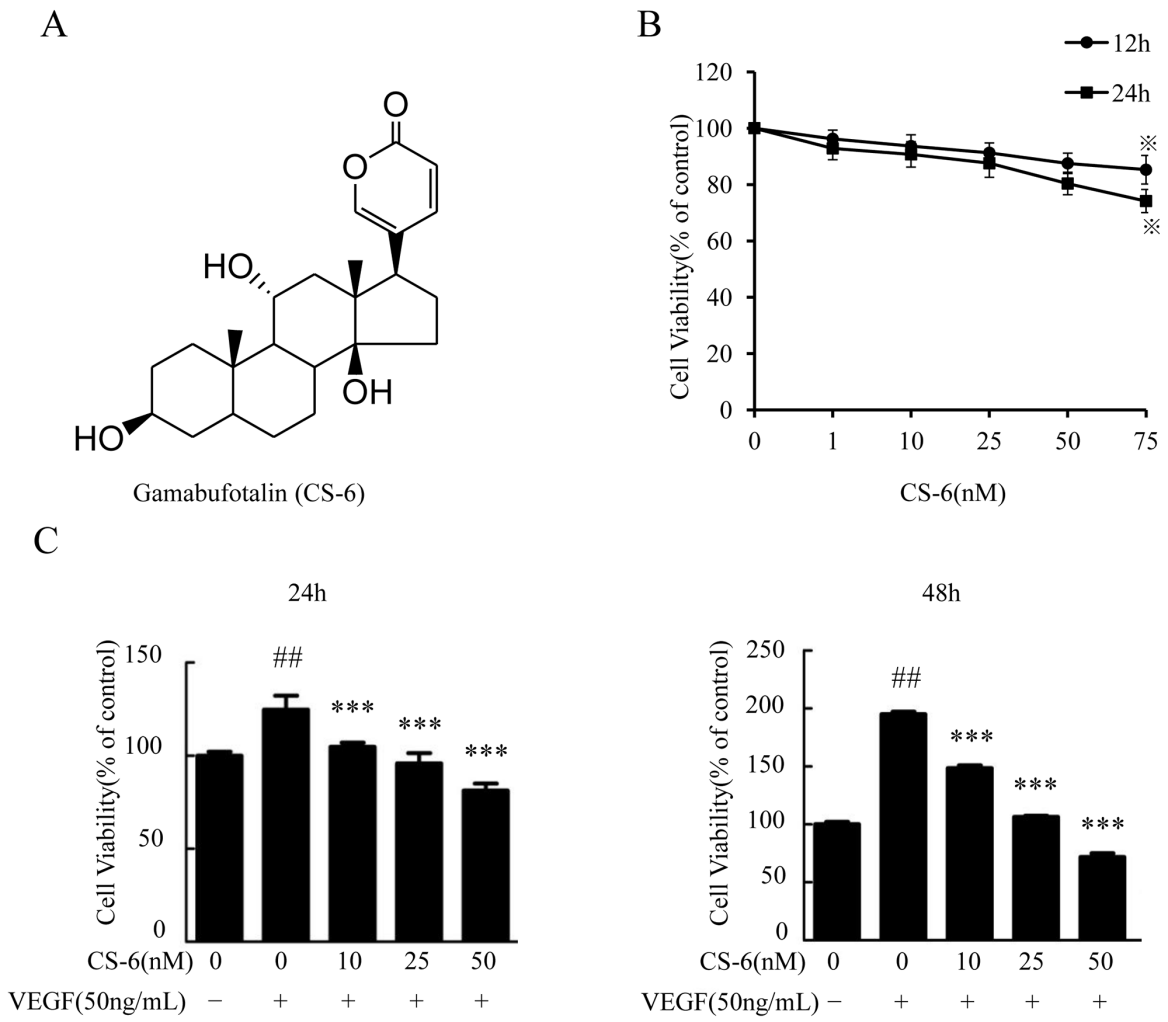
CS-6 inhibits VEGF-induced proliferation of HUVECs **A.** Chemical structure of gamabufotalin (CS-6). **B.** Viability inhibition of CS-6 on HUVECs under normal culture condition. HUVECs were exposed to CS-6 at the indicated doses and times, and viability was measured by MTT assay. Data were represented as percentage of vehicle-treated control. **C.** CS-6 inhibits the proliferation of VEGF-induced HUVECs. HUVECs were treated with CS-6 with or without 50 ng/mL VEGF for 24 h or 48 h, and viability was measured by MTT assay. Three independent experiments were performed (*p<0.05, CS-6-treated group vs. DMSO group; ##p < 0.01, VEGF-treated group vs. Solvent; ***p < 0.001, VEGF and CS-6-treated group vs. VEGF-treated group).

As an importance of proliferation for endothelial angiogenesis and tumor growth [35], we firstly investigated the influence of CS-6 (Fig. 1A) in human endothelial cells proliferation. After treatment for HUVECs with a range of CS-6 (0, 1, 10, 25, 50 and 75 nM) for 12 h and 24 h and cell viability was determined by MTT assay. The results revealed CS-6 has mild inhibition for HUVECs proliferation and showed no obvious cytotoxicity at low concentration (Fig. 1B).

We further determined whether CS-6 inhibited VEGF-induced HUVECs cell growth using MTT assay. As shown in Fig. 1C, the number of HUVECs stimulated with VEGF for 24 h and 48 h increased about 1.25 folds and 1.9 folds, respectively. These results showed that CS-6 could inhibit VEGF-induced cell growth in a dose-dependent and time-dependent manner; however we observed a greater inhibition by the CS-6 in VEGF stimulated HUVECs proliferation in comparison with absence of VEGF (Fig. 1C). Taken together, our data showed that CS-6 was a potent inhibitor of VEGF-activated endothelial cell proliferation.

### CS-6 inhibits VEGF-induced endothelial cell migration and invasion

**Figure 2:**
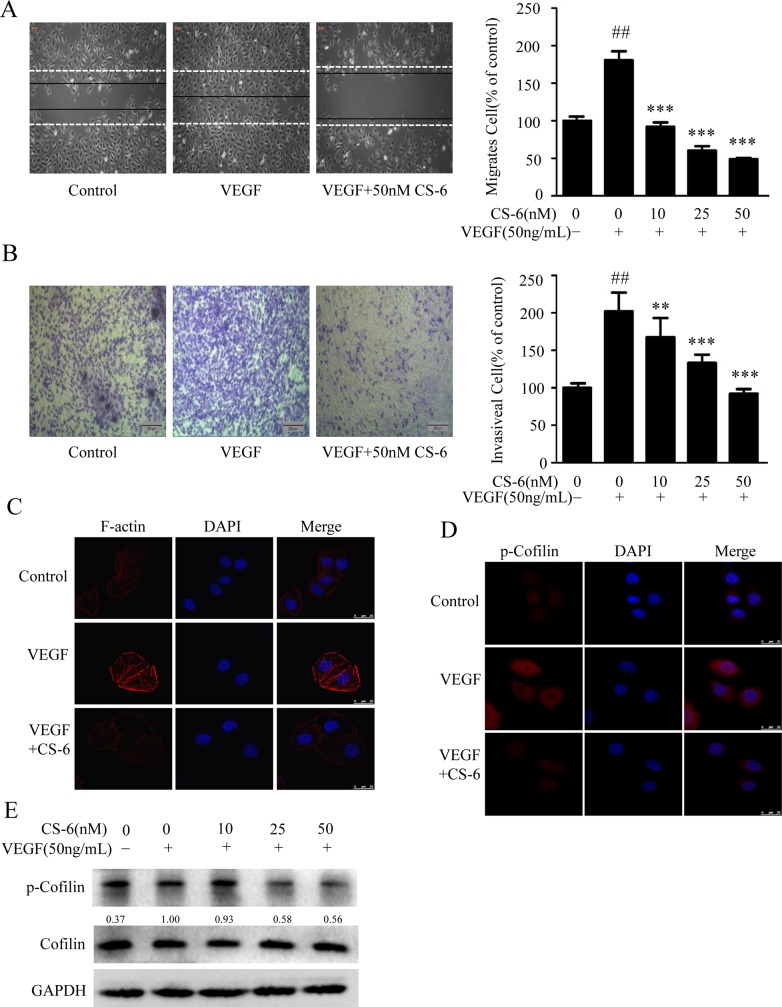
CS-6 inhibits VEGF-induced endothelial cell migration and invasion **A.** CS-6 inhibits HUVECs migration induced by VEGF in wound healing assay. HUVECs were plated, scratched and then incubated with CS-6 with or without 50 ng/mL VEGF. Cell migration was measured by manual counting. Original magnification, 100× **B.** CS-6 inhibits HUVECs invasion in Transwell assay. HUVECs were plated in Transwell pre-coated with matrigel. Cell migrated to the bottom of the membrane were counted by using an inverted microscope. Original magnification, 40× **C.** CS-6 suppressed VEGF-induced stress fibre formation in endothelial cells. HUVECs were exposed to 50 nM CS-6 for 0.5 h, and then stimulated with or without VEGF for 15 min. F-actin of cells was visualized by dyLightTM 554 palloidin staining and imaged by Leica confocal microscopy. **D.** CS-6 inhibited VEGF-induced cofilin phosphorylation in HUVECs using immunofluorescences staining with specific antibody for phosphorylated cofilin. E. CS-6 inhibited VEGF-induced cofilin phosphorylation and activation in Western Blotting assay. Three independent experiments were performed (##p < 0.01, VEGF-treated group vs. Solvent; **p < 0.01, VEGF-treated group vs. VEGF and CS-6-treated group; ***p < 0.001, VEGF-treated group vs. VEGF and CS-6-treated group).

Cell migration and invasion are essential for endothelial cells during angiogenesis. We next investigated the effects of CS-6 on cell migration and invasion by wound healing assays and Transwell assays, respectively. The results showed that CS-6 significantly inhibited the migrating and invasive properties of VEGF-induced endothelial cells in a dose-dependent manner (Fig. 2A and 2B).

As cell cytoskeleton and stress fibre formation are key cellular events involved in cell migration, we further investigated the effects of CS-6 on these aspects. Confocal image analysis of individual cells revealed that VEGF caused a robust induction of stress fibre formation which was inhibited by 50 nM CS-6 (Fig. 2C). As activation of cofilin is an essential component of actin polymerization and depolymerization, and significantly affects cell cytoskeleton reorganization, we examined the effects of CS-6 on VEGF-induced phosphorylated cofilin using immunofluorescence techniques. Results showed in Fig. 2D demonstrate that 50nM CS-6 reduced VEGF-induced cofilin phosphorylation and activation. Furthermore, western blotting further confirmed the phosphorylation and activation of cofilin were reduced by CS-6 in a dose-dependent manner (Fig. 2E). These results suggest that CS-6 inhibited VEGF triggered HUVECs motility by affecting cofilin activity and cell stress fibre formation.

### CS-6 suppresses VEGF-induced anti-apoptosis of HUVECs

**Figure 3:**
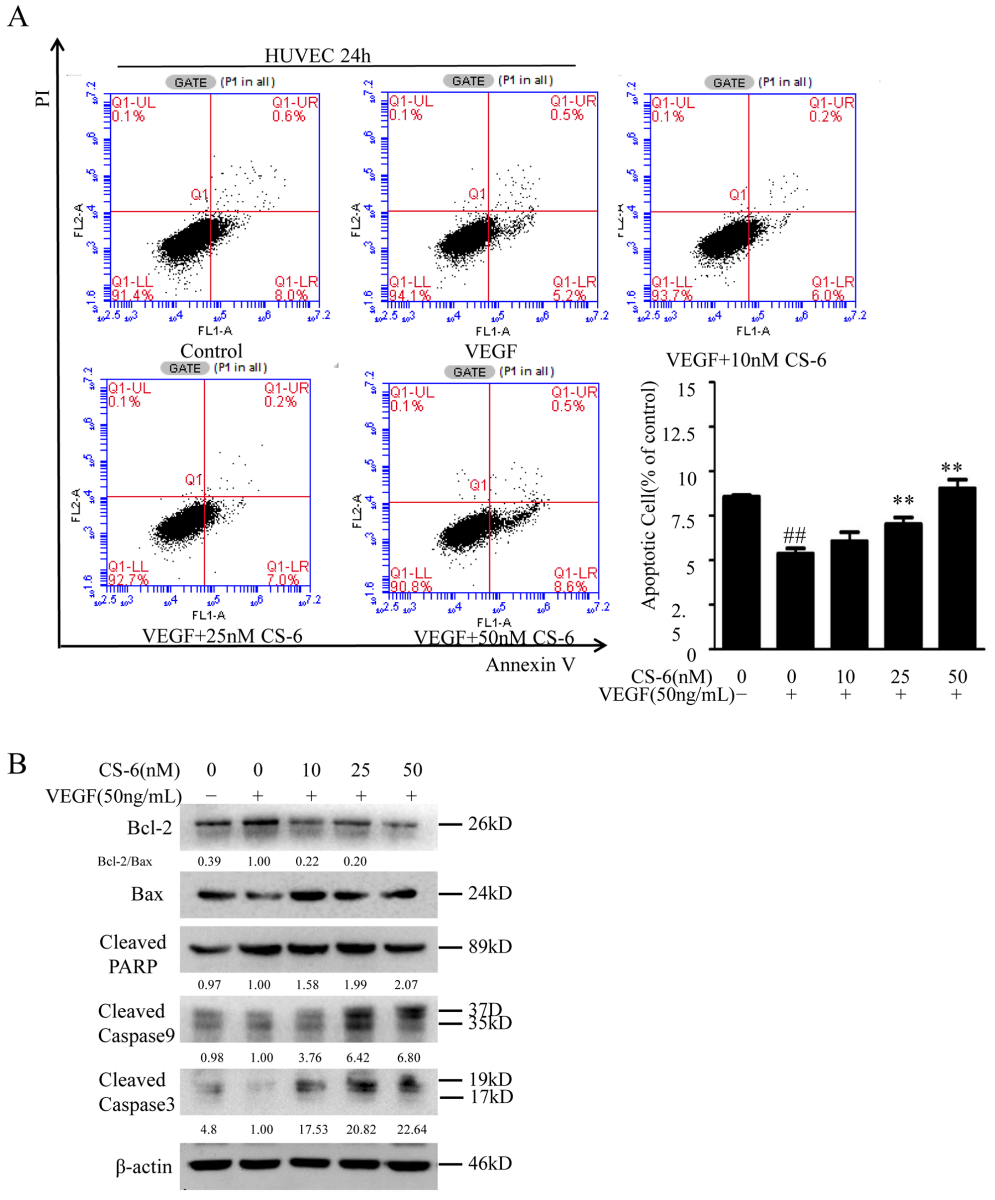
CS-6 suppresses VEGF-induced anti-apoptosis in HUVECs HUVECs were incubated for 24 h with indicated CS-6 after 24 h of serum starvation, along with or without VEGF. The cell apoptosis rate was determined by a FACS analysis **A.** and the levels of Bcl-2, Bax, cleaved caspase-3/9 and cleaved PARP proteins were nalyzed by Western blot **B.** The apoptosis is represented by relative percentages of apoptotic cells versus that in DMSO-treated cells. Three independent experiments were performed (##P < 0.01, VEGF group vs. control group; **P<0.01, ***P < 0.001, VEGF- and CS-6-treated group vs. VEGF-treated group).

Consistent with the findings of other investigators that VEGF caused a marked decrease in the apoptosis of HUVECs induced by serum deprivation, as indicated by a VEGF-dependent decrease in cell-surface annexin V binding [36]. We next investigated the effects of CS-6 on VEGF caused anti-apoptosis in HUVECs using Annexin V/propidium iodide assay. As shown in Fig. 3A, the experimental group stimulated with VEGF decreased the apoptosis of HUVECs induced by serum deprivation from 8.6% in control cells to 4.3%, however, co-treated with indicated doses CS-6 and VEGF can dramatically increase cell apoptosis in HUVECs compared with VEGF group, which suggests that CS-6 inhibits VEGF-induced anti-apoptosis. We also detected the expression of three key pro-apoptotic proteins (PARP and caspase-3/9), as well as Bcl-2 and Bax protein by Western blot analysis. CS-6 could markedly increase the expression levels of the cleaved caspase-3/9 and PARP proteins, and reduce the ratio of Bcl-2/Bax as compared with the VEGF group (Fig. 3B).

### CS-6 inhibits VEGF-induced angiogenesis *in vitro* and *ex vivo*

**Figure 4:**
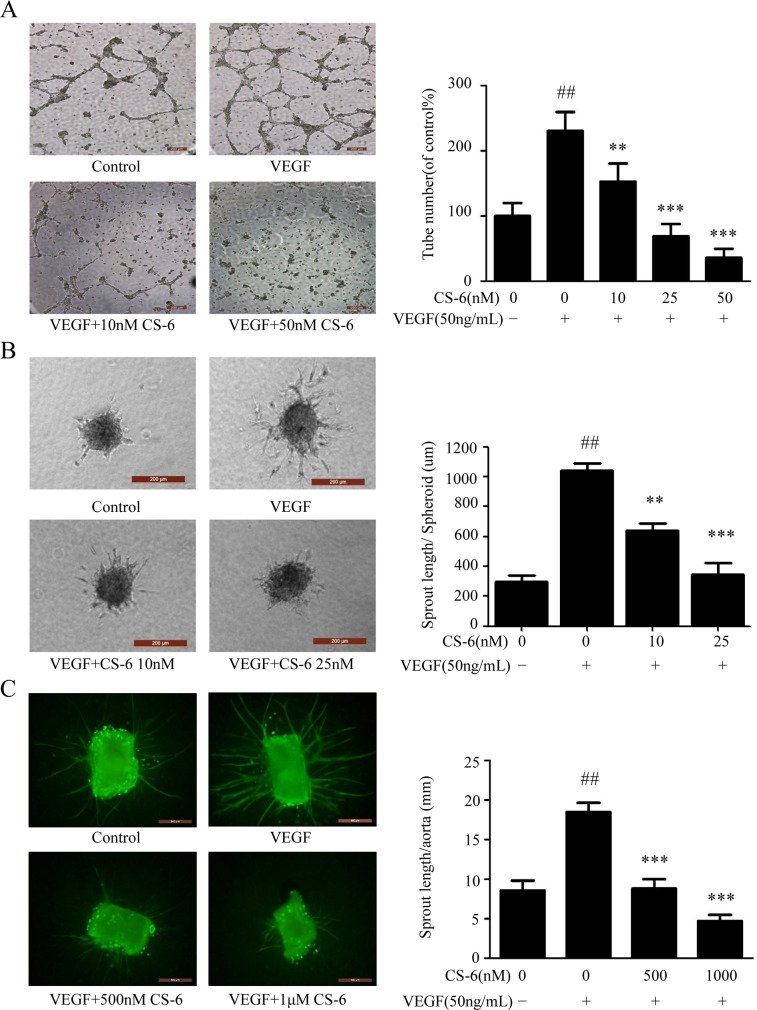
CS-6 inhibits VEGF-induced angiogenesis *in vivo* and *ex vivo* Effect of CS-6 on **A.** tube formation on Matrigel (Original magnification, 50×) and **B.** sprouting from modified human endothelial cell spheroids (Original magnification, 200×). Experiments were performed with or without VEGF and indicated CS-6 doses. **C.** Endothelial sprouting from aortic rings from C57/BL6 mice. Additional VEGF (50 ng/mL) and CS-6 were added and the endothelial sprouts were allowed to develop over 9 days, and then staining with FITC-Lectin (green) Original magnification, 50×. Three independent experiments were performed (##p < 0.01, VEGF-treated group vs. no VEGF-treated group; **p < 0.01, VEGF-treated group vs. VEGF- and CS-6-treated group; ***p < 0.001, VEGF-treated group vs. VEGF- and CS-6-treated group).

One of the important steps during neo-angiogenesis is the formation and merging of tubes produced by endothelial cells forming a complex network of vessels and capillaries [37, 38]. Furthermore, we examined whether CS-6 inhibited VEGF-induced HUVECs tube formation using an *in vitro* angiogenesis tube formation assay. As shown in Fig. 4A, HUVECs were placed on the surface of Matrigel, and VEGF (50 ng/mL) significantly enhanced (p<0.001) the endothelial capillary like structures comparing with solvent treated cells. However, the VEGF effects were blocked by CS-6 treatment in the doses indicated. These data indicated that CS-6 would efficiently impair VEGF induced tube formation in HUVECs.

We further examined the VEGF also induced endothelial cell sprouting in a modified spheroid assay; however, this response was significantly impaired by CS-6 in endothelial cells (Fig. 4B). Moreover, capillary formation assay was carried out through mouse dorsal aortas in *ex vivo*. After nine days of culture in matrigel, we observed a significant increase in endothelial sprouts from VEGF treated mouse aorta rings, while the microvessel sprouting was inhibited in a dose-dependent manner by CS-6 treatment (Fig. 4C).

### CS-6 inhibits the activation of VEGFR-2 in HUVECs

**Figure 5:**
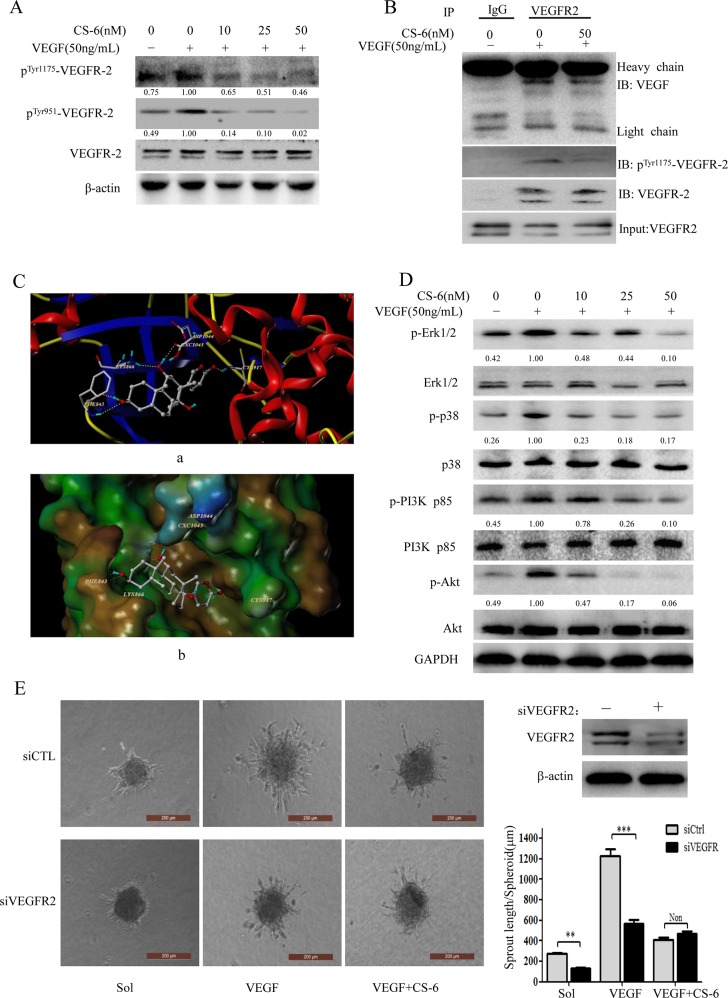
CS-6 inhibits the activation of VEGFR-2 kinase in HUVECs and has no effect on VEGF binding to VEGFR-2 **A.** CS-6 inhibits the phosphorylation of VEGFR-2. HUVECs were pre-treated with CS-6 for 6 h, and then stimulated with 50 ng/mL VEGF for 1 h. **B.** CS-6 did not interfere with VEGF binding to VEGFR-2. Whole-cell extracts were collected and analyzed by Co-IP assay and Western blotting using antibodies against VEGF, VEGFR-2 and pTyr1175-VEGFR-2. Three independent experiments were performed (##p < 0.01, VEGF-treated group vs. no VEGF-treated group; **p < 0.01, VEGF-treated group vs. VEGF and CS-6-treated group; ***p < 0.001, VEGF-treated group vs. VEGF and CS-6-treated group). **C.** The docking stereo view of CS-6 with ATP binding site of VEGFR-2. (a) Interactions of CS-6 and VEGFR-2 are delineated by ribbon structure. (b) MOLCAD surface representation of the ATP binding site of VEGFR-2. Hydrogen bonds are displayed as yellow dashed lines, and the participating amino acid residues are marked. **D.** CS-6 suppresses activation of VEGFR-2-mediated signaling pathway. HUVECs were pre-treated with CS-6 for 6 h, and then stimulated with 50 ng/mL VEGF for 1 h. Whole-cell extracts were extracted for Western blotting analysis. **E.** Effect of CS-6 on sprouting from modified human endothelial cell spheroids or VEGFR2-knockdown human endothelial cell spheroids (Original magnification, 200×).

It is well appreciated the great contribution of VEGF/VEGFR-2 axis in regulating endothelial mitogenesis as well as migration [39]. Previous studies indicated that blockage of VEGFR-2 activity could significantly limit tumoral neo-angiogenesis process [40]. Considering the CS-6 antagonizing VEGF induced angiogenic effects, we assess the influence of CS-6 on VEGF triggered VEGFR2 phosphorylation and as data shown in Fig. 5A. As shown in Fig. 5A, the addition of exogenous VEGF induced VEGFR-2 phosphorylation in two different phosphorylation sites (Tyr951 and Tyr1175), and VEGFR-2 phosphorylation at both Tyr951 and Tyr1175 was specifically suppressed by treatment with CS-6 without affecting the overall VEGFR-2 expression level. As expected, CS-6 limited VEGF-induced phosphorylation of VEGFR-2 in a dose-dependent manner. We hypothesized that CS-6 might bind to VEGFR-2 and subsequently affect the interaction between VEGF and VEGFR-2. To verify this possibility, next, the computer docking simulations of the interaction of CS-6 with VEGFR-2 were carried out. Molecular docking studies predicted that CS-6 would bind at the ATP binding site of VEGFR-2. As shown in Fig. 5C (a), CS-6 forms five hydrogen bonds with the ATP binding pocket of the VEGFR-2 kinase domain. The CO group at the lactonic ring of CS-6 forms a hydrogen bond with the backbone NH of Cys917. The OH group at the C14 position forms strong hydrogen bonds with the backbone at Cxc1045 and Lys 866 simultaneously. Moreover, 3-OH accepts two hydrogen bonds with the CO and NH residues of Phe843. The result of MOLCAD surface modeling indicated that the lactonic ring of CS-6 extends into the deep hydrophobic cavity of the ATP-binding pocket of VEGFR-2 (Fig. 5C, b). Moreover, we carried out Co-IP experiment to further explore whether CS-6 could interfere with the basis for VEGF-VEGFR-2 interaction. As shown in Fig. 5B, both VEGF and VEGFR-2 were detected in the resulting precipitates, indicating that VEGF was associated with VEGFR-2. The expression of phosphor^Tyr1175^-VEGFR-2 was obviously reduced with the treatment of CS-6, which was consistent with the result of Western blotting analysis (Fig. 5B). These results showed that CS-6 had little effect on VEGF-VEGFR-2 interaction, but suppressed the activation of VEGFR-2.

### CS-6 suppresses activation of VEGFR-2-mediated signaling pathway

VEGFR-2 activation initiates complex signaling networks with distinct and overlapping functions. We further examined whether CS-6 inhibited VEGFR-2-mediated signaling pathways. As shown in Fig. 5D, CS-6 significantly suppressed the activation of VEGFR-2 downstream signaling molecules such as PI3K/Akt and MAPK pathways, which are critical for VEGF/VEGFR-2-mediated angiogenesis. To be specific, p-Erk1/2 and p-p38 were enhanced by VEGF treatment while the expression level of Erk1/2 and p38 remained unchanged, and CS-6 inhibited the phosphorylation of Erk1/2 and p38 without affecting total Erk1/2 and p38 expression levels. CS-6 also reduces phosphorylation levels of Akt at Ser473, which is a well-known downstream target of VEGFR-2, however, has no effect on total Akt level. Moreover, the action of CS-6 on PI3K p85 was also examined.

Furthermore, in order to verify the anti-angiogenesis effect of CS-6 is via VEGFR2, we next knocked down VEGFR2 by siRNA, and performed endothelial cell spheroids sprouting assay. As shown in Fig. 5E, knockdown of VEGFR2 by siRNA considerably inhibited endothelial cell sprouting in sol group and VEGF group compared with non-siRNA knockdown in a modified spheroid assay, however, there was no obvious difference in CS-6-treatment group. These data indicated that the anti-angiogenesis effect of CS-6 was through VEGFR2 in HUVECs.

### CS-6 inhibits VEGF-induced blood vessel formation *in mice*

**Figure 6:**
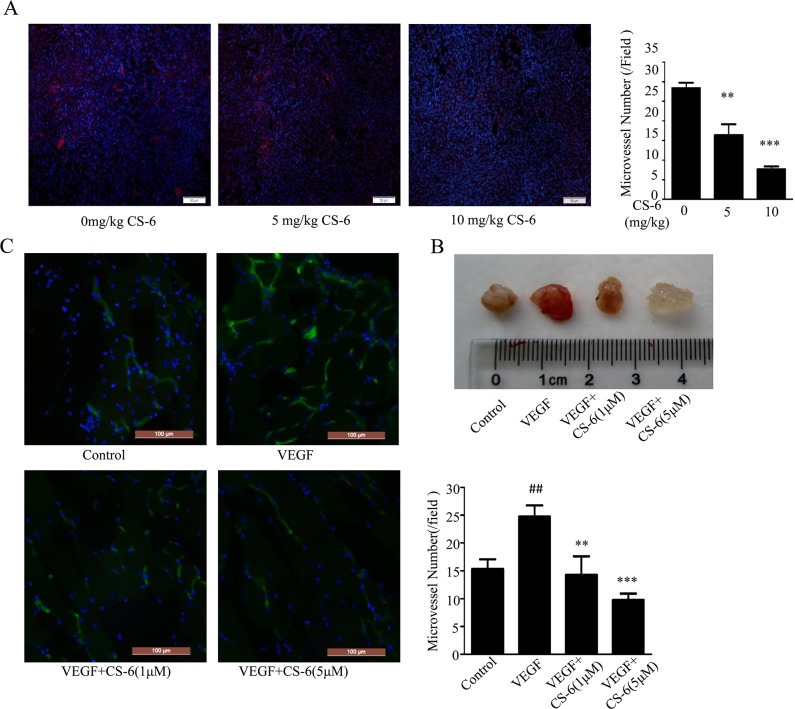
CS-6 inhibited angiogenesis *in vivo* **A.** Neutral formalin fixed tumor samples were prepared from animals and analyzed by immunohistochemical staining with VE-cadherin, and examined under a microscope. (**P < 0.01, ***P < 0.001, significant differences between CS-6 treatment groups and control group. Red is displayed as VE-cadherin protein). **B.** The axillary fossa of 6-week-old C57/BL/6 mice was injected with 500 mL of matrigel with 250 ng VEGF and 80 unit of heparin. After 9 days, the matrigel plugs were harvested and photographed. **C.** The matrigel plugs were fixed, sectioned and stained with FITC-Lectin. (##p < 0.01, VEGF-treated group vs. no VEGF-treated group; **p < 0.01, VEGF-treated group vs. VEGF- and CS-6-treated group; ***p < 0.001, VEGF-treated group vs. VEGF- and CS-6-treated group. Green is displayed as FITC-Lectin).

To confirm our findings *in vivo*, we assess the impacts of CS-6 on endothelial angiogenesis in lung cancer xenografts model as well as martrigel plugs. model. Our previous study has shown that CS-6 inhibited the growth of lung cancer xenografts in nude mice [34]. Herein, we further examined the vessel density in lung cancer tissue sections visualized using VE-cadherin staining (Red) under immunofluorescent microscope. We chose VE-cadherin because of its modulation of angiogenesis [41], and its expression could reflect the microvessel density in the tissues. Direct inhibition of VE-cadherin function could impact other signaling functions such as modulation of VEGF receptor signaling. As shown in Fig. 6A, the micro-vessel density identified by VE-cadherin staining was significantly decreased with CS-6 treatment, as compared with the vehicle group. However, we could not exclude possibility that the CS-6 treatment impaired vessel density in the human xenograft was due to the tumor suppressive role of CS-6. To clarify this issue, we further applied matrigel plug assay. After 7 days, matrigel plugs containing VEGF excised from mice were dark red and filled with blood vessels. In contrast, matrigel plugs containing both VEGF and CS-6 were light yellow. The group treated with 5 μM CS-6 was nearly transparent (Fig. 6B). We then used FITC-isolectin and DAPI to stain and quantify the number of functional vessels in the matrigel plugs. As shown in Fig. 6C, fewer vessels were observed in the matrigel plugs treated with both VEGF and CS-6 than in those plugs treated with VEGF alone. These results indicate that CS-6 can significantly inhibit angiogenesis *in vivo*.

Taken all together, our results showed that gamabufotalin (CS-6) exerted its anti-angiogenic effect by suppressing VEGFR-2 activation.

## DISCUSSION

Our previous study showed that gamabufotalin (CS-6), in the nanomolar range, markedly reduced cell proliferation and migration of NSCLC cells [34]. However, little is known about the inhibitory effects of CS-6 on angiogenesis and associated molecular mechanism. Angiogenesis is important for tumor growth, maintenance and invasion. Tumor growth is initially fed by nearby blood vessels, and new blood vessels are required to support the growth when the tumor size exceeds a certain size [42]. Therefore, Neo-angiogenesis is the critical step in the development and progression of most of the human cancers, and anti-angiogenic therapy of cancer treatment is a new method for cancer treatment.

In previous study, we found that CS-6 reduced cell growth in several human lung cancer cell lines (IC_50_ was about 55 nM), while had no adverse effect on human normal lung cell line (HLF cells). The interesting observation may suggest that CS-6 would be more favor to affect tumor cell viability, while whether or not it potentially influencing endothelial angiogenesis during tumor development is totally unknown. Herein, we presented in current study that CS-6 showed minor inhibitory effects (IC_50_ was more than 200 nM) on HUVECs, a similar phenomena observed in previous tests of normal human lung cells; however, CS-6 would remarkably inhibit VEGF induced endothelial growth, suggesting it may interact VEGF signaling as well corresponding endothelial bio-function. Indeed, we found that CS-6 could inhibit angiogenesis *in vitro*, *ex vivo* and *in vivo*, and suppress key steps related to angiogenesis, including proliferation, survival, migration and angiogenesis in endothelial cells. However, we noticed the anti-angiogenic effective doses of CS-6 among cell cultures (10, 25 and 50 nM), aortic tissue cultures (500 and 1000 nM) as well animal levels (1000 and 5000 nM) are different, which may be resulted from varied growth environments or complex background. In order to exclude the possibility that toxicity of CS-6 would give raise to false positive phenomenon on endothelial angiogenesis, we well controlled these issues. For example, the doses of CS-6 used in cell cultures were no clear toxic effects determined by MTT assay; in aortic ring tissue cultures, we did not observed CS-6 increased apoptotic endothelial cells by PI staining. Further investigation showed that CS-6 inhibited angiogenesis via suppressing of VEGF-induced VEGFR-2 phosphorylation and activation by targeting the ATP-binding site of VEGFR-2. Here, to the best of our knowledge, it is the first time to reveal that CS-6 inhibits angiogenesis both in *vitro* and *in vivo*.

It has been well documented that Cyclooxygenase-2 (COX-2), as one of the VEGF/VEGFR-2 signaling client proteins, plays a central role in the modulation of angiogenesis, and our previous study found CS-6 suppressed COX-2 expression in non-small cell lung cancer (NSCLC) cells. Therefore, we investigate whether CS-6 inhibit angiogenesis was associated COX-2 expression. We discovered CS-6 would not affect COX-2 expression in HUVECs in basal culture condition; however, it was able to specifically reduce VEGF-induced elevation of COX-2 in endothelial cells (Data not shown). It demonstrated CS-6 performs clearly different mechanism in regulating COX-2 expression in endothelial cell as a downstream event of VEGF-VEGFR2 signaling with the transcriptional regulation in NSCLC.

Tumor growth depends on angiogenesis and anti-angiogenic therapy is a new therapeutic approach for cancer treatment. Previous research indicates that VEGF/VEGFR-2 signaling pathway is the main cascade involved in angiogenesis growth, and Erk1/2 and PI3K/Akt pathways could regulate VEGF expression [43]. VEGFR-2 activation stimulates complex signaling networks with distinct and overlapping functions, including MAPK and PI3K/Akt pathways, which play important roles in vascular endothelial cell growth, survival and migration. In this study, we showed that CS-6 suppressed PI3K/Akt and MAPK signaling pathways through inhibiting VEGFR-2 activation by targeting the ATP-binding site.

VEGF and its high-affinity receptor VEGFR-2 are the most widely studied factors in angiogenesis. Targeting of VEGFR-2 is an intriguing strategy in the anti-angiogenic therapy of tumors [44], which is essential for the functions of vascular endothelial cells. Thus, we studied whether CS-6 could inhibit VEGF-induced VEGFR-2 activation. In the present study, CS-6 was shown to inhibit VEGF-induced tyrosine phosphorylation of VEGFR-2 in a dose-dependent manner. Moreover, computational docking showed that CS-6 occupied the deep hydrophobic pocket in the ATP-binding site of VEGFR-2. In this modeling analysis, five hydroxyl groups bound to the ATP-binding pocket of VEGFR-2 via forming five hydrogen bonds. The CO motif at the lactonic ring of CS-6 forms hydrogen bond with Cys917 of the hinge region of VEGFR-2 [45]. Residue Cys917 is a crucial amino acid for ligand reorganization and binding on the ATP site as reported. The hydroxyl group at the C-14 position of cs6 bound to the ATP-binding pocket of VEGFR-2 via forming two hydrogen bonds with residues Lys866 and Cxc1045 of the polar region. These hydrogen bond interactions were important for improvement the binding affinity of CS-6 to VEGFR-2 [46]. The additional hydrogen bond interactions of CS-6 with Phe843 stabilized its occupation of the hydrophobic pocket of VEGFR-2. These above data suggest that CS-6 may inhibit angiogenesis by targeting VEGFR-2.

## MATERIALS AND METHODS

### Chemicals and reagents

Gamabufotalin (CS-6) was isolated from *ChanSu* in our lab, which was secreted from the postauricular and skin glands of *Bufo bufo gargarizans Cantor* [34]. In our study, CS-6 was dissolved in DMSO as a 100 μM stock solution and diluted in the relevant assay media and kept at −20°C. CS-6 was diluted in culture medium to obtain the desired concentration, which was stable in the dilution with DMSO concentration less than 1‰.

### Antibodies and other materials

3-(4, 5-dimethylthiazol-2-yl)-2,5-diphenyltetrazolium (MTT), dimethyl sulfoxide (DMSO), M199 and Lectin from Bandeiraea simplicifolia (Griffonia simplicifolia) were purchased from Sigma Chemical Co. (St. Louis, MO). Recombinant human VEGF_165_ and human basic fibroblast growth factor (bFGF), were obtained from PeproTech Inc. Recombinant human EGF was purchased from ProSpec Company. Matrigel were purchased from BD Bioscience (San Diego, CA). The primary antibodies for VEGF, VEGFR-2, p^Tyr1175^-VEGFR-2, p^Tyr951^-VEGFR-2, p-p38, p-ERK, p38, ERK, Akt, p-Akt, PI3K p110α, PI3K p85, p-PTEN, p-Colifin, Colifin, cleaved-caspase3/9, cleaved-PARP and all the secondary antibodies were acquired from Cell Signaling Technology (Cell Signaling Technology, Inc, USA). The primary antibodies for GAPDH, β-actin, Bax and Bcl-2 were obtained from Proteintech Group (Proteintech Group, Inc., USA). Annexin V-FITC apoptosis detection kit was purchased from Roche (Indianapolis, IN). Control siRNA (5′-uucuuccgaacgugucacgutt-3′) and siRNA against human VEGFR2 (5′-gcggcuaccaguccggauatt-3′) were synthesized by genepharm. Trypsin and DMEM/F12 were obtained from HyClone, and fetal bovine serum (FBS) was obtained from Gibico. M199 Medium was bought from LIFE. siRNA and all other chemicals were purchased from Sigma Chemical Co. unless otherwise specified.

### Cell culture

Primary human umbilical vein endothelial cells (HUVECs) were isolated from human umbilical vein as described[47]. HUVECs were cultured in M199 containing 10% fetal bovine serum (FBS), 25 U/mL heparin, 5 ng/mL bFGF and 10 ng/mL EGF, The cells were cultured in a humidified atmosphere of 5% CO_2_ at 37°C.

### siRNA transfection

After one hour serum free starvation in M199 medium with antibiotics, and HUVECs were transfected with control or siRNA against human VEGFR2 were by lipofectamine 3000 (Invitrogen) according to manufactory instruction.

### Cell proliferation assay

HUVECs viability was measured by MTT assay. Briefly, HUVECs were seeded at 8 × 10^3^ cells/well in 96-well plates, and allowed to adhere to obtain 80% confluent monolayer. The medium was replaced with normal growth medium containing CS-6 (0, 1, 10, 25, 50 and 75 nM). After 12 h or 24 h incubation, the growth of cells was measured at 490 nm using an *EnSpire*® Multimode Plate Reade (Perkin Elmer, USA). The effect of CS-6 on VEGF-induced cell viability was measured by MTT assay. HUVECs (6 × 10^3^ cells/well) were respectively treated with or without VEGF (50 ng/mL) or various concentration of CS-6 (0, 10, 25 and 50 nM) for 24 h and 48 h, and then cell growth was measured. In control cells, equivalent amount of DMSO was added as vehicle. The number of viable cells was presented relative to untreated controls.

### Wound healing migration assay

HUVECs were allowed to grow into full confluence in 6-well plates. A line of HUVECs was then scraped away in each well using a pipette tip after 6h of serum starvation. Subsequently, cells were washed twice to remove detached cells. Fresh M199 medium containing different concentrations of CS-6 (0, 10, 25 and 50 nM) or vehicle, with or without 50 ng/mL VEGF was added to the scratched monolayers. Images were taken using a Leica DM 14000B microscope after 12 h incubation. The migrated cells were observed from three randomly chosen fields and quantified by manual counting. Inhibition percentage was expressed as percentage of the untreated cells (100%).

### Endothelial cell transwell invasion assay

The motility of HUVECs was performed in 24-well transwell plates [48]. The upper surface of polycarbonate filters with 8 μm pores was coated with 75 μL Matrigel (Matrigel: M199=1:3, without growth factors) and incubated for 0.5 h at 37°C for gelling. Then, Cells were seeded into the upper chambers at a density of 5 × 10^4^ cells per chamber, the bottom chamber were filled with 600 μL M199 with 10% FBS supplemented with VEGF (50 ng/mL) or vehicle. Both top and bottom chamber contained the same concentrations of CS-6. After 24 h incubation, non-invasive cells on the upper membrane surfaces were removed by wiping with cotton swabs. Invaded cells were fixed with methanol and stained with 0.1% Crystal Violet Staining Solution. The membrane was dried in the air. Images were taken using a Leica DM 14000B microscope. Cell invasion counted in five independent areas per membrane. The results were the means calculated from five replicates of each experiment.

### Tube formation assay

HUVECs [1 × 10^4^ in 50 μL M199 medium with 0.1% BSA containing CS-6 (0, 10, 25, 50 nM)] were seeded on Ibitreat angiogenesis slides (Ibidi, Martinsried, Germany) pre-coated with 10 μL Matrigel, and the formation of tubular like structure was recorded by a Leica DM14000B microscope at 6 hours or 24 hours.

### Endothelial cell spheroids sprouting assay

Spheroids were generated by gravity as described [47]. Subsequently, spheroids were harvested by 5 min centrifugation at 1000 rpm and embedded into a collagen gel (containing collagen (1 mg/mL); 1x M199 (sigma); 0.22% NaHCO_3_) with pH at 7.4 adjusted by 0.1 N NaOH. The spheroid-containing gel was rapidly transferred into a 24-well plate and allowed to polymerize at 37°C incubators for 30 min, M199 medium with or without VEGF and CS-6 was applied on top of the gel. Spheroids sprouts were evaluated by measuring the cumulative length of all capillary like sprouts using a Leica DM14000B microscope. At least 5 randomly selected spheroids per experimental group and experiment were analyzed. And sprout length was measured with Image J software.

### Aortic ring assay

Aorta from C57/BL6 mice were removed, cleaned and approximate 1mm aortic rings were embedded in a collagen gel (collagen type I, BD Biosciences) in a 48 well plate, After gel polymerization, DMEM/F12 medium (HyClone) supplemented with 2.5% mouse serum. Additional VEGF (50 ng/mL) and CS-6 (500 nM and 1000 nM) were added and the endothelial sprouts were allowed to develop over 9 days. Thereafter, the samples were fixed (4% paraformaldehyde) and endothelial cells were visualized using FITC-Lectin (Sigma) staining under immunofluorescent microscope. The results were the means calculated from five replicates of each experiment.

### Matrigel plug assay

All animals were maintained, and animal experiments were done in SPF Laboratory Animal Center at Dalian Medical University. The animals used in this research were 6 weeks old male C57/BL6 mice (three mice per group). Matrigel (0.5 mL/plug) containing 250 ng VEGF and 80 units heparin with various concentrations of CS-6 (1 or 5 μM) were injected (S.C.) into near the axillary fossa of mice. Matrigel mixed with medium alone was used as a negative control. After 9 days of implantation, the matrigel plugs were removed and the surrounding tissues were trimmed. The matrigel plugs were embedded with O.C.T. Compound (Sakura Finetek USA, Inc.). Ten-micron sections were stained by FITC-Lectin (Sigma) staining. The number of blood vessels in high power field (HPF, magnification) was counted under immunofluorescent microscope. The results were the means calculated from five replicates of each experiment.

### Microvessel density assay by VE-cadherin staining

Human lung xenografts samples were from our previous study [34]. 4μm sections were stained with VE-cadherin (1:200, Santa Cruz, sc-6458) and DAPI (1:5000). The endothelial staining was photographed under immunofluorescent microscope and vessel density was evaluated by Image J software. The results were shown as Mean ± SD from at least 5 tumor samples for each group. The tumor sections were from three treatment groups that each contained five mice.

### Molecular modeling

Docking studies were performed to explore the potential binding interactions between CS-6 and VEGFR-2. The compound CS-6 was optimized using the semi-empirical PM3 method with the Polak-Ribie're conjugate gradient algorithm with an RMS gradient of 0.001 kcal mol^−1^ Å^−1^ as convergence criterion. The optimized structure was further docked into the active site of VEGFR-2 (PDB Code: 1YWN). The crystallographic ligand was extracted from the active site, and the residues within a 6.5 Å radius around the VEGFR-2 molecule were defined as the active site. The Surflex-Dock program was used for the docking calculations with default parameters. MOLCAD surfaces were generated for visualizing the binding mode of the docked protein–ligand complexes.

### Co-immunoprecipitation (Co-IP)

In order to determine the effect of CS-6 on the interaction between VEGFR-2 and VEGF, HUVECs were incubated in M199 with 0.5% FBS for 6 h followed by treating with CS-6 for 4 h, followed by 50 ng/mL VEGF_165_ treatments for 1 h. Briefly, cell extract proteins (500 μg) were pre-cleared by adding 1 μg normal rabbit IgG and 40 μL protein A/G-Agarose beads. Together, they were incubated at 4°C for 1 h with gentle agitation and centrifuge. The supernatants were collected and incubated with a specific rabbit anti-VEGFR-2 antibody at 4°C overnight with gentle rotary agitation. The immune complexes were pulled down by protein A/G agarose beads (Santa Cruz Biotechnology) and washed with ice-cold PBS buffer containing proteinase inhibitor for 3 times. After final wash, the immune complexes were released by boiling in 2 × electrophoresis sample buffer for 5 min, followed by western blotting analysis with rabbit anti-VEGFR-2, rabbit anti-phospho^Tyr1175^-VEGFR-2 and mouse anti-VEGF antibodies.

### Cell apoptosis analysis

Cell apoptosis analyses were carried out by fluorescence-activated cell sorter (FACS). After treatment, the cells were harvested by 0.25% trypsin and washed by PBS buffer and then stained simultaneously with FITC-labeled annexin V and PI. Stained cells were analyzed using FACS Accuri C6.

### F-actin staining and immunofluorescence analysis

HUVECs were seeded onto coverslips in a 6-well plate. The cells were treated with or without 50 μM CS-6 for 1h and then were stimulated with 50 ng/mL VEGF for 15min. After that, the cells were fixed in 4% paraformaldehyde for 20 min, and then permeabilized with 0.2% Triton X-100 for 5 min at room temperature. Actin filaments were stained by phalloidin-FITC for 20 min, and nuclei were detected by DAPI. For phosphorcofilin immunofluorescence, cells were blocked with 5% BSA in PBS for 1 h. After washing three times with PBS, cells were incubated with antibody against phospho-cofilin overnight at 4°C, and then with secondary antibodies for another 1 h at room temperature. Next, cell nuclei were stained by DAPI for 5 min. The slides were examined with a Leica DM 14000B confocal microscope.

### Western blotting

To determine the effects of CS-6 on VEGFR-2-dependent signaling cascade, starved HUVECs were treated with CS-6 for 6 h, followed by 50ng/mL VEGF_165_ treatments for 1 h. Cells were lysed with an appropriate cold lysis buffer (Beyotime Biotechnology, China) supplemented with proteinase inhibitor cocktail (Sigma, USA). Protein concentrations were determined using a BCA protein assay kit (Beyotime Biotechnology, China). Proteins (50 μg) were applied to 8% to 12% SDS-PAGE gels and transferred onto a PVDF membrance (Millipore, USA). The membranes were incubated with specific primary antibodies. Protein bands were detected using an enhanced chemiluminescence method. Bands were normalized with GAPDH or β-actin as an internal control. Similar experiments were performed at least three times.

### Statistical analysis

Data are represented as mean ± SD obtained for at least three independent experiments. Statistical significance was determined by one-way ANOVA. P<0.05 was considered to be a statistically significant difference. SPSS 17.0 software was used for all statistical analysis. The mean density of the interest bands (where mentioned) was determined using Scion Image software.

## CONCLUSION

We demonstrated for the first time that CS-6 as a natural bufadienolide, exhibited the significant anti-angiogenesis effect by inhibiting proliferation, migration, invasion, tube formation and enhancing apoptosis of human umbilical vein endothelial cells in a dose-dependent manner. Moreover, its molecular mechanisms inhibiting angiogenesis are mainly mediated through suppressing the VEGF/VEGFR-2-mediated signaling cascades by targeting ATP binding site, inactivating PI3K/Akt and MAPK pathways. From the above, these findings strongly demonstrate that CS-6 could serve as a potential candidate for anti-angiogenic therapy and a potent inhibitor of VEGFR-2-activated.
